# Targeting glioma stem cells enhances anti-tumor effect of boron neutron capture therapy

**DOI:** 10.18632/oncotarget.9355

**Published:** 2016-05-13

**Authors:** Ting Sun, Yanyan Li, Yulun Huang, Zizhu Zhang, Weilian Yang, Ziwei Du, Youxin Zhou

**Affiliations:** ^1^ Neurosurgery and Brain and Nerve Research Laboratory, The First Affiliated Hospital of Soochow University, Suzhou, Jiangsu, China; ^2^ Beijing Capture Tech Co., Ltd, Beijing, China

**Keywords:** boron neutron capture therapy, glioma stem cells, target, CD133, bioconjugate

## Abstract

The uptake of ^(10)^boron by tumor cells plays an important role for cell damage in boron neutron capture therapy (BNCT). CD133 is frequently expressed in the membrane of glioma stem cells (GSCs), resistant to radiotherapy and chemotherapy, and represents a potential therapeutic target. To increase ^(10)^boron uptake in GSCs, we created a polyamido amine dendrimer, conjugated CD133 monoclonal antibodies, encapsulating mercaptoundecahydrododecaborate (BSH) in void spaces, and monitored the uptake of the bioconjugate nanoparticles by GSCs *in vitro* and *in vivo*. Fluorescence microscopy showed the specific uptake of the bioconjugate nanoparticles by CD133-positive GSCs. Treatment with the biconjugate nanoparticles resulted in a significant lethal effect after neutron radiation due to efficient and CD133-independent cellular targeting and uptake in CD133-expressing GSCs. A significantly longer survival occurred in combination with the biconjugate nanoparticles and BSH compared with BSH alone in human intracranial GBM models employing CD133-positive GSCs xenografts. Our data demonstrated that this bioconjugate nanoparticle targets human CD133-positive GSCs and is a potential boron agent in BNCT.

## INTRODUCTION

Boron neutron capture therapy (BNCT) is a high-dose tumor-selective radiotherapy, which utilizes non-radioactive isotope ^(10)^boron (^10^B) to capture thermal neutrons with high probability leading to nuclear reaction of ^10^B(*n,α*)^7^Li. Treating tumors with high linear-energy-transfer (LET) alpha and ^7^Li particles is effective biologically. As the range of these particles in tissue is limited to 10–14 μm, the use of short-range radiation ensures that adjacent normal tissues are spared from radiation-induced damage [1]. In theory, high LET radiation kills anoxic and quiescent cells, as well as oxygenated and proliferative cells [2, 3]. However, successful BNCT requires selective targeting and delivery of adequate ^10^B to all tumor cells (∼ 20 μg/g weight or ∼10^9^ atoms/cell). Previous studies confirmed ^10^B uptake by proliferative cells targeted for killing with thermal neutrons [4–6].

Glioblastoma (GBM) is the most aggressive class of brain tumors. Although a transient response to therapy is often observed, tumor recurrence invariably occurs within the tissue subjected to intensive combination of surgical resection, radiotherapy and chemotherapy [7, 8]. Currently, there is no cure for GBM, because the surgeon cannot effectively resect this diffuse tumor. Resistant cells that escape radiotherapy- and chemotherapy-induced cell death stay dormant for extended periods after treatment but eventually re-enter the cell cycle, leading to tumor re-growth. In a consensus publication that prospectively identified cells with increased tumorigenicity, this subpopulation was termed “Glioma Stem Cells” (GSCs) [9, 10]. Lack of effective absorption of existing boron agents by GSCs leads to glioma cell survival and tumor recurrence after BNCT. Therefore, the development of alternative formulations for boron agents targeting GSCs is a key requirement for BNCT.

To develop polyamido amine (PAMAM) dendrimer carriers encapsuling boron agent conjugated with ligand for targeted delivery, mercaptoundecahydrododecaborate (B12H11SH(2-), BSH) was incorporated into the PAMAM dendrimer as a boron agent. A CD133 antibody was conjugated to PAMAM dendrimer to bind the ligand of CD133 membrane antigen. The biconjugate of PD-CD133/BSH was specifically absorbed by CD133-positive (CD133+) GSCs, and GSCs were killed by BNCT *in vitro* and *in vivo*. PD-CD133/BSH was characterized by its effective uptake by CD133+ GSCs, and the combination of PD-CD133/BSH and BSH extended survival in a nude mouse model of orthotopic glioma after neutron radiation.

## RESULTS

### Identification of biconjugate

**Figure 1:**
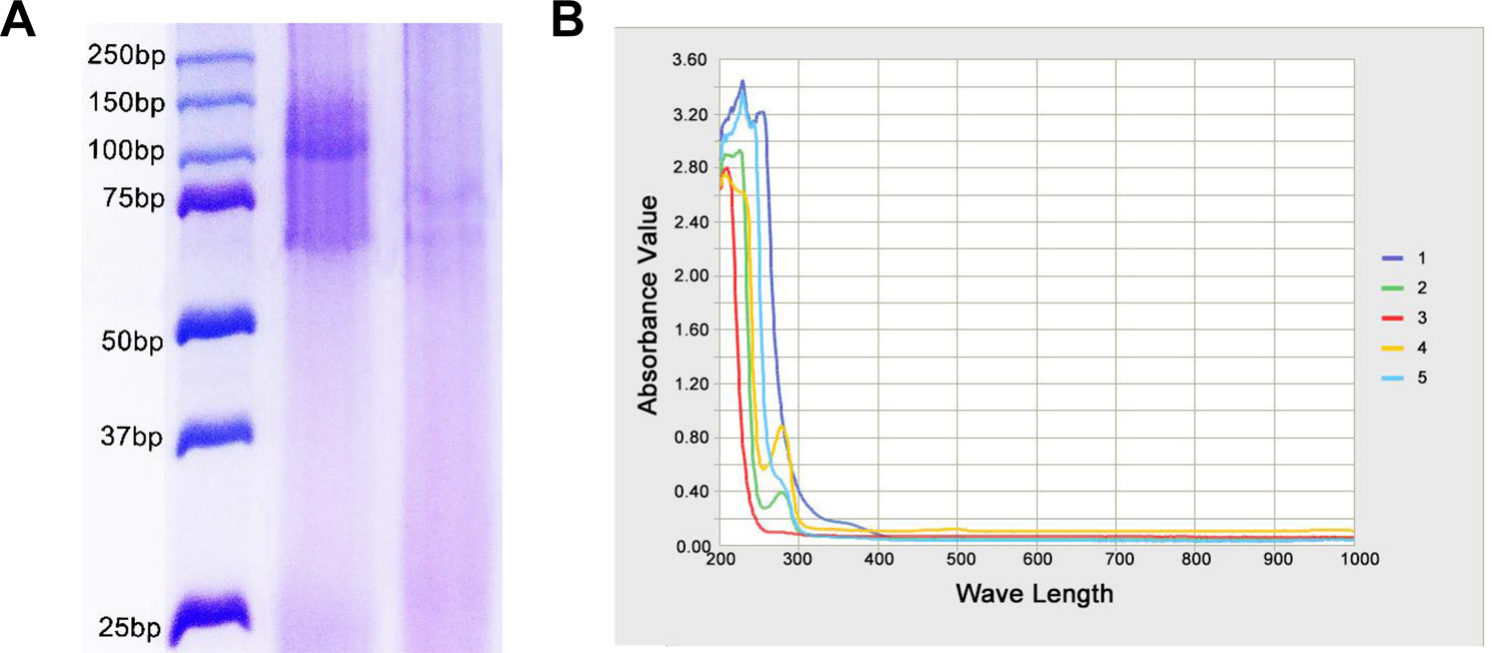
Confirmation of mAb-CD133 conjugation to PAMAM (**A**) The conjugated production of PAMAM and mAb-CD133 was observed by SDS-PAGE. Lane 1. marker Lane2. PD-CD133 Lane.3 mAb-CD133 (**B**) Ultraviolet-visible spectra of bioconjugated production PD-CD133. Characteristic absorbance peak at about 260 nm and 280 nm indicated the presence of BSH and CD133 antibody, respectively. 1.BSH 2.mAb-CD133 3.PAMAM 4.PD-CD133 5.PD-CD133/BSH.

The conjugation of PAMAM to anti-CD133 monoclonal antibody (mAb-CD133) was confirmed by SDS-PAGE. A 70 KDa band was seen in the mAb-CD133 group, compared with a 100 KDa band in the conjugate of PAMAM and mAb-CD133 (PD-CD133) group, and no band appeared in about 70 KDa range (Figure 1A). The results indicated successful conjugation and purification. Conjugation of mAb-CD133 and PAMAM was aided by ultraviolet-visible spectra of all bands scanned. The biconjugated PD-CD133 was observed at an absorption peak of 280 nm as mAb-CD133, but no peak appeared in PAMAM. Compound BSH showed an absorption peak at approximately 260 nm. Therefore, PD-CD133/BSH has absorption peaks at 260 nm and 280 nm, which were not distinct (Figure 1B). One mAb-CD133 molecule was conjugated to each molecule of PAMAM dendrimers, which was determined by SDS-PAGE, ultraviolet-visible spectra and Ellman assay. The concentration of the biconjugated PD-CD133 was calculated as 300 μM using a mAb-CD133 standard curve.

### Encapsulating efficiency and loading capacity of biconjugate

The results of encapsulating efficiency (EE%) and loading capacity (LC%) show that EE% of BSH was (76.2 ± 4.5)% in PAMAM and (73.8 ± 6.7)% in PD-CD133 as drug carrier. LC% of BSH was (13.56 ± 0.88)% in PAMAM and (2.99 ± 0.12)% in PD-CD133 as drug carrier. The BSH concentration was 6.5 mM in PAMAM solution and 7.0 mM in PD-CD133 solution. No significant differences in EE % and BSH concentration were observed between PAMAM and PD-CD133. However, the LC% of BSH in PD-CD133 was significantly decreased compared with that of PAMAM (*P* < 0.01) due to the presence of mAb-CD133.

### PD-CD133/BSH targets CD133+ cells in surgical section sample of GBM

**Figure 2:**
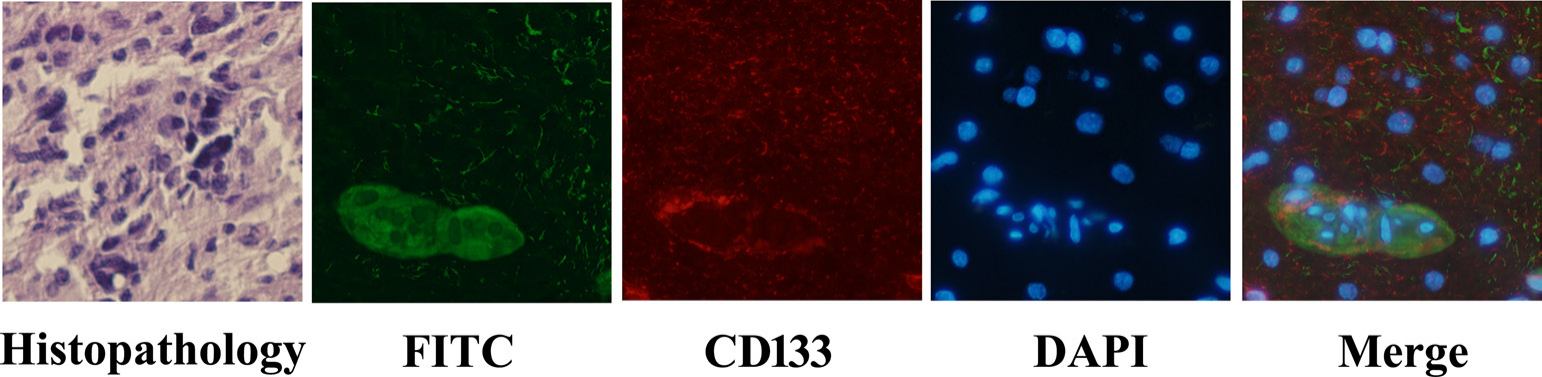
PD-CD133/BSH uptake in surgical section sample of GBM GBM from patients showed Grade IV by histopathology. Green fluorescence was derived from PD-CD133/BSH, and red fluorescence was CD133 stain using immunofluorescence. Cell nuclei was stained blue by 4′,6-diamidino-2-phenylindole (DAPI) (×400).

Fluorescent microscopy was carried out after glioblastoma tissues were incubated with 0.1 μM PD-CD133/BSH for 12 h. PD-CD133/BSH was indicated by green fluorescence, and cells expressing CD133 membrane antigen were stained by red fluorescence. As showed in Figure 2, PD-CD133/BSH was specifically absorbed by CD133+ cells, which suggested that PD-CD133/BSH was internalized by cells expressing CD133 antigen in the membrane targeted by CD133 antibody. Cells with no CD133 antigen expression absorbed little PD-CD133/BSH and no green fluorescence was observed. PD-CD133 has targeting characteristics similar to CD133 membrane antigen.

### Identification of sorted GSCs

**Figure 3:**
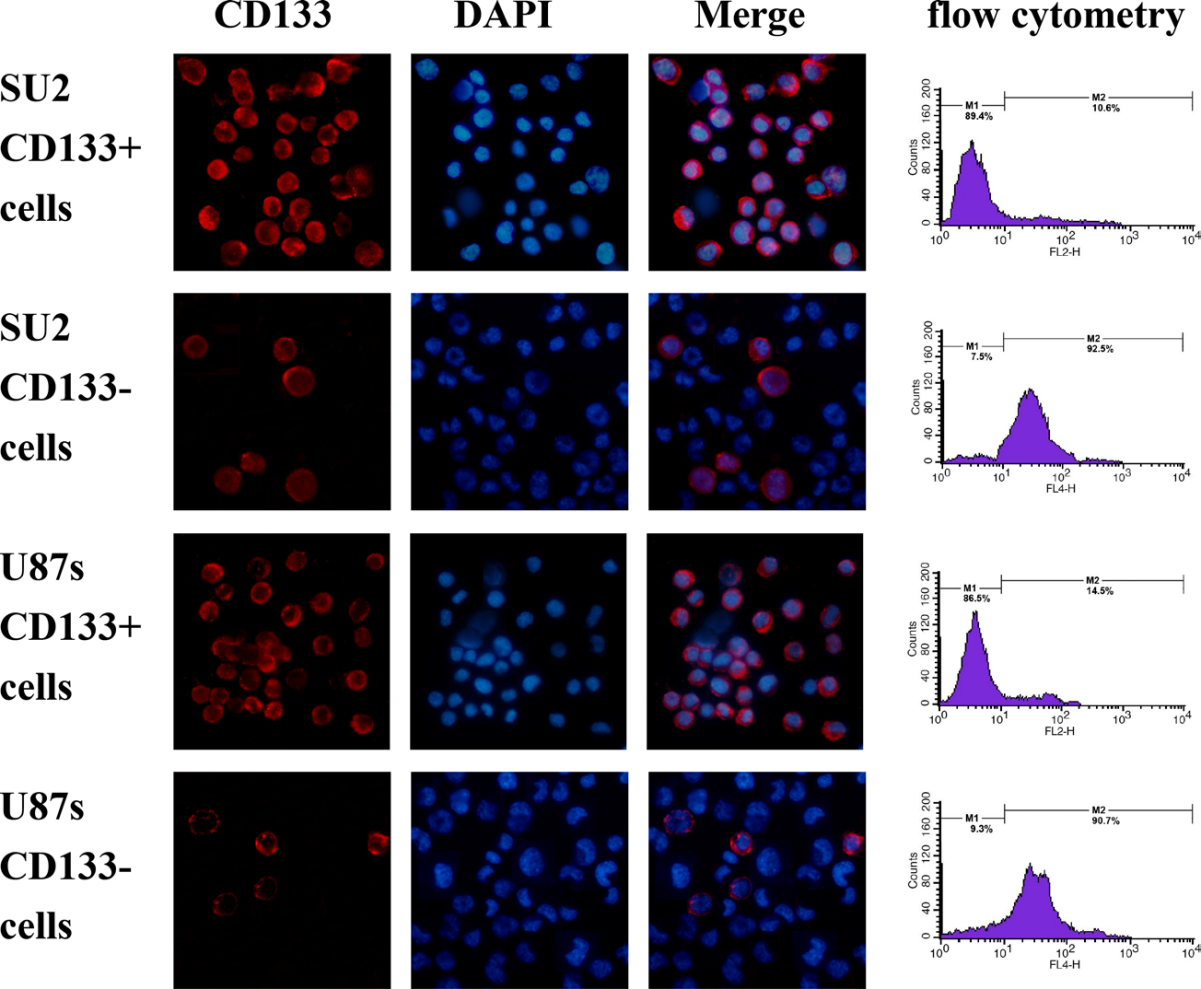
Identification of sorted GSCs The percentage of CD133-positive cells in sorted GSCs analyzed by flow cytometry, and fluorescence images of sorted GSCs, immunostained with antibodies against CD133, were captured with fluorescence microscope (×400).

In order to detect the percentage of SU2 and U87s cells with CD133+ surface marker and sorting efficiency, a quantitative analysis of CD133 positive cells was performed using flow cytometry. After sorting by magnetic beads, the two cell lines were separated into two groups, respectively. In the CD133+ group, 92.5% SU2 or 90.7% U87s cells positively expressed the CD133 marker, and 89.4% SU2 or 86.5% U87s cells did not express the CD133 marker in the CD133− group (Figure 3). Immunofluoresence staining results showed that a majority of both SU2 and U87s cells strongly expressed glioma stem cell marker CD133 in CD133+ group and did not express CD133 marker in CD133- group, which mediate self-renewal and proliferation of stem cells (Figure 3).

### Uptake efficacy and ^10^B concentration

**Figure 4:**
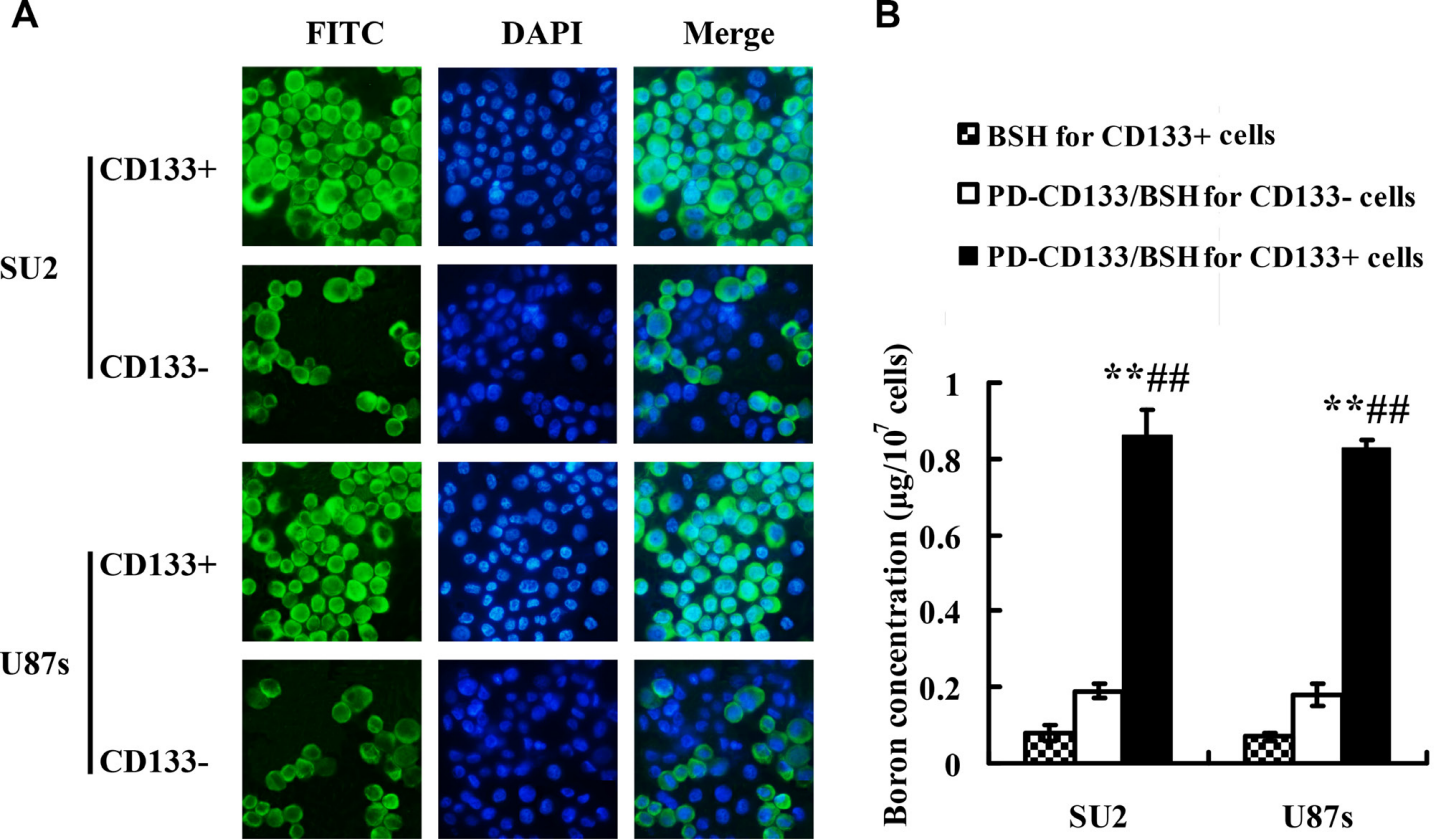
Uptake efficacy for PD-CD133/BSH and ^10^B concentration (*n* = 3) (**A**) Uptake efficacy of sorted CD133+ and CD133− GSCs observed in fluorescence microscope with 0.1 μM PD-CD133/BSH for 12 h (×400). (**B**) Concentration of boron in cultured GSCs incubated with 0.1 μM PD-CD133/BSH solution or 2.2 μM BSH for 12 h. Boron accumulation in both SU2 and U87s CD133+ cells cultured with PD-CD133/BSH was significantly higher than in the CD133− cells (*P* < 0.01) and BSH treatment (*P* < 0.01). **P < 0.01 vs. PD-CD133/BSH for CD133− cells; ^##^*P* < 0.01 vs. BSH for CD133+ cells.

To evaluate the uptake efficiency of PD-CD133/BSH, the CD133+ and CD133− SU2 cells were cultured with different concentrations of PD-CD133/BSH for different periods. Uptake efficiency of PD-CD133/BSH [(95.7 ± 4.6)%] was significantly increased after 12 h when 0.1 μM PD-CD133/BSH was added to CD133+ SU2 cells compared with CD133- SU2 cells [(38.5 ± 4.7)%] (*P* < 0.01). Simultaneously, uptake efficiency of [(91.8 ± 7.6) %] and [(29.4 ± 3.2) %] occurred in CD133+ and CD133− U87s cells, respectively (Figure 4A), which was significantly different (*P* < 0.01) (Table 1). The concentration of ^10^B in the CD133+ SU2 and U87s cells supplemented with PD-CD133/BSH was 0.86 ± 0.07 μg/10^7^ cells (5.18 × 10^9^ atoms in each cell) and 0.82 ± 0.02 μg/10^7^ cells (4.94 × 10^9^ atoms in each cell), respectively, which was higher than in CD133− SU2 (0.19 ± 0.02 μg/10^7^ cells, 1.14 × 10^9^ atoms in each cell) and U87s (0.18 ± 0.03 μg/10^7^ cells, 1.08 × 10^9^ atoms in each cell) cells (*P* < 0.01) (Figure 4B).

**Table 1:** Uptake efficiency of PD-CD133/BSH in CD133+ and CD133− GSCs (%)

Treatment		SU2		U87s	
		CD133+ cells	CD133− cells	CD133+ cells	CD133− cells
Concentration (μM) 12 h	0.05	31.8 ± 1.7^*^	12.4 ± 0.9	29.7 ± 2.8^*^	10.5 ± 0.4
	0.10	95.7 ± 4.6^**^	38.5 ± 4.7	91.8 ± 7.6^**^	29.4 ± 3.2
	0.50	96.5 ± 7.6^**^	45.2 ± 2.6	92.5 ± 8.1^**^	44.7 ± 4.2
	1.00	98.7 ± 8.5^**^	62.7 ± 6.8	93.1 ± 5.3^**^	63.1 ± 4.9
Time (h) 0.1 μM	1	6.5 ± 0.9	7.1 ± 0.4	7.1 ± 0.6	5.8 ± 0.4
	6	48.5 ± 7.7^*^	27.1 ± 3.1	50.3 ± 4.9^**^	28.5 ± 0.7
	12	95.7 ± 4.6^**^	38.5 ± 4.7	91.8 ± 7.6^**^	29.4 ± 3.2
	24	97.2 ± 13.6^**^	47.6 ± 6.4	95.8 ± 9.1^**^	50.6 ± 7.2

Cells Positive cells were observed using fluorescence microscope, and cells with green fluorescence were identified as positive cells.

* *P* < 0.05

** *P* < 0.01 vs. CD133− cells at the same comcentration and time point

### Clonogenic survival after neutron radiation

**Figure 5:**
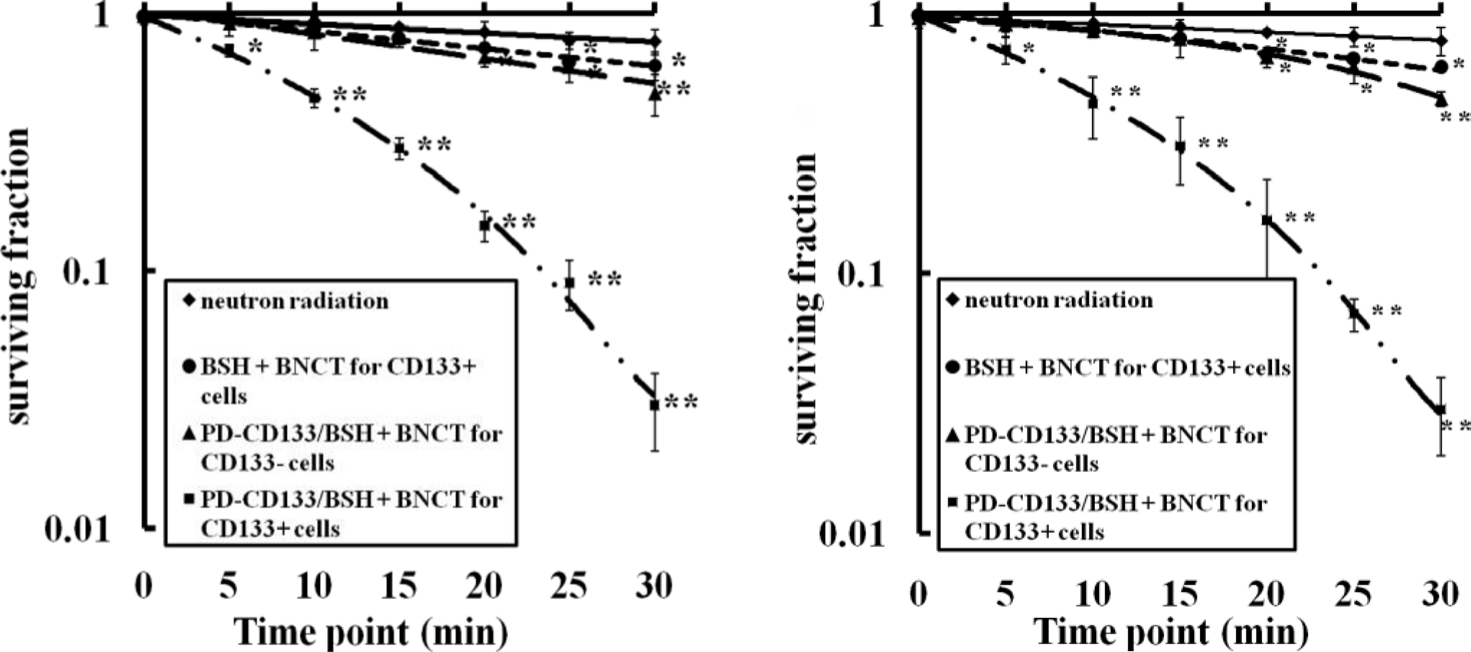
Survival curves of SU2 (A) or U87s (B) cells after BNCT determined by clonogenic assay Neutron radiation: the CD133+ cells treated without ^10^B agent were irradiated with neutron, BSH + BNCT for CD133+ cells: the CD133+ cells treated with 2.2 μM BSH for 12 h were irradiated with neutron, PD-CD133/BSH + BNCT for CD133+ cell: the CD133+ cell treated with 0.1 μM PD-CD133/BSH for 12 h were irradiated with neutron, PD-CD133/BSH + BNCT for CD133− cell: the CD133- cell treated with 0.1 μM PD-CD133/BSH 12 h were irradiated with neutron. *P < 0.05, **P < 0.01 vs. non-irradiated cells.

Cell survival was investigated using a clonogenic assay after exposure to neutron radiation. SU2 and U87s cell surviving curves were obtained after neutron irradiation according to the linear quadratic model (Figure 5). In all the groups, surviving fractions were decreased in a dose-dependent manner. The CD133+ cells in the PD-CD133/BSH BNCT group were suppressed more strongly than in neutron irradiation, BSH BNCT and PD-CD133/BSH BNCT for CD133− cells group. For SU2 cells, the surviving fraction (0.72 ± 0.04) of PD-CD133/BSH BNCT in CD133+ cells group was decreased after irradiation for 5 min compared with non-irradiation (0.96 ± 0.02) (*P* < 0.05), and the decrease (0.47 ± 0.04) was more significant with irradiation for 10 min (*P* < 0.01). Significant differences were found in PD-CD133/BSH BNCT for CD133− cells group compared with BSH BNCT group when irradiated for 20 min (*P* < 0.05) and BSH BNCT group when irradiated for 25 min (*P* < 0.05). Similar results occurred in the U87s cells group except for the significant decrease (*P* < 0.05) in the BSH BNCT group following irradiation for 20 min.

### Imaging studies showed PD-CD133/BSH targeting to CD133+ cells *in vivo*

**Figure 6:**
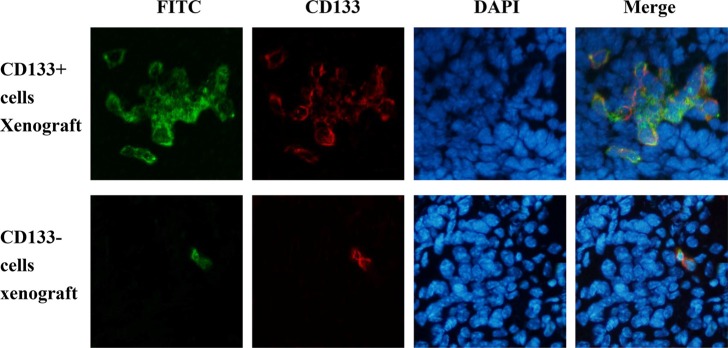
PD-CD133/BSH was specifically localized in CD133+ SU2 cells in orthotopic glioma xenografts (**A**) Tumor pathologic examination confirmed the formation of intracranial glioblastoma derived from SU2 cells. (**B**) Green fluorescence was derived from PD-CD133/BSH, and red fluorescence represented CD133 stain using immunofluorescence (×400).

The biodistribution of PD-CD133/BSH after i.v. injection in SU2 glioma-bearing mice is shown in Figure 6. Microscopic examination of the brains of tumor-bearing animals revealed targeting features of PD-CD133/BSH in the cerebral hemisphere. PD-CD133/BSH uptake by the cells was indicated by green fluorescence associated with the FITC unit from CD133 antibody. Proportions of CD133+ cells in brain between CD133+ and CD133− SU2 cells transplanted mice were different, and differences in biodistribution of PD-CD133/BSH were clearly observed as a series of green fluorescence from PD-CD133/BSH and red fluorescence from CD133 stain. As determined by fluorescence of individual mice, PD-CD133/BSH was specifically localized in CD133+ SU2 cells.

### Boron biodistribution in mice bearing glioma xenografts

Boron concentrations in tumor tissue, ipsilateral brain, contralateral normal brain tissues and blood were detected and shown in Table 2. After BSH injection for 1 h, selective boron accumulation in CD133+ glioma cell xenografts was observed. Boron levels in brain tissues and blood were in the undetectable range (< 0.5 μg/g) in mice, which were injected with PD-CD133/BSH at 12 h. The boron concentration was increased in xenografts derived from CD133+ (7.7 ± 2.9 μg/g) compared with CD133− (2.4 ± 0.4μg/g) cells after 12 h of PD-CD133/BSH injection (*P* < 0.01). The mean boron concentration was (25.7 ± 5.8) μg/g after 12 h following administration of PD-CD133/BSH in combination with BSH 1 h in CD133+ SU2 xenografts. There was no significant difference in boron concentration between CD133+ and CD133− (22.6 ± 3.8 μg/g) cell xenografts when combined with treatment of PD-CD133/BSH and BSH (*P* > 0.05).

**Table 2:** Boron concentrations in tumor, ipsilateral brain, contralateral brain and blood after injection of single BSH, PD-CD133/BSH or combination into mice with CD133+ or CD133− glioma cells xenografts (mean ± SE, μg/g)

Glioma xenografts	Boron Agents	Tumor tissue	Ipsilateral brain	Contralateral brain	blood
CD133+ cells	BSH 1 h	21.2 ± 1.9	14.6 ±1.9	12.5 ± 1.7	9.2 ± 1.6
	PD-CD133/BSH 12 h	7.7 ± 2.9^**^	——	——	——
	PD-CD133/BSH 12 h + BSH 1 h	25.7 ± 5.8	15.9 ± 2.1	12.8 ± 4.6	9.4 ± 2.8
CD133− cells	BSH 1 h	20.8 ± 3.4	14.2 ± 1.1	13.1 ± 2.2	8.3 ± 2.1
	PD-CD133/BSH 12 h	2.4 ± 0.4	——	——	——
	PD-CD133/BSH 12 h + BSH 1 h	22.6 ± 3.8	14.7 ± 1.6	12.6 ± 2.8	8.9 ± 2.3

Boron content was calculated by boron solution standard curve using ICP-AES. These values were obtained from mice, implanted glioma xenografts with CD133+ or CD133− SU2, injected with PD-CD133/BSH 12 h (3.5 μg ^10^B) or BSH (100 mg/kg b.w.) 1 h alone or in combination. Boron concentrations were detected in determined in ipsilateral brain and contralateral brain of tumor implanted mice after tumor excision.

** *P* < 0.01 vs. PD-CD133/BSH 12 h for CD133− cells glioma xenografts

### Therapeutic response of glioma-bearing mice following BNCT

**Figure 7:**
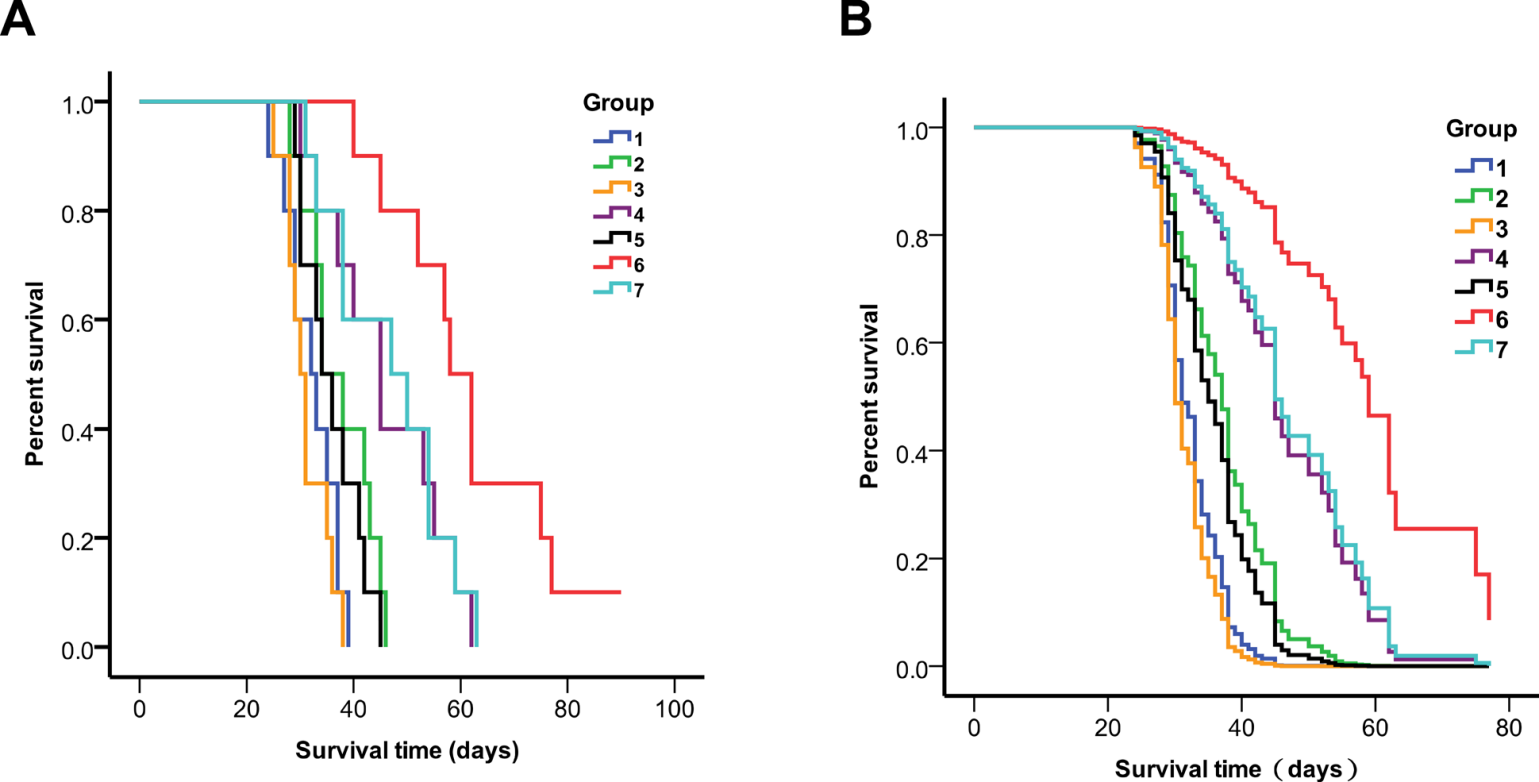
Survival analysis of CD133+ or CD133- SU2 cell implanted mice 1 untreated control for CD133+ cells, 2. neutron radiation control for CD133+ cells, 3. PD-CD133/BSH + BSH for CD133+ cells, 4. BSH + BNCT for CD133+ cells, 5. PD-CD133/BSH + BNCT for CD133+ cells, 6. PD-CD133/BSH + BSH + BNCT for CD133+ cells, 7. PD-CD133/BSH + BSH + BNCT for CD133− cells. (**A**) Kaplan-Meier survival plots; (**B**) Cox survival plots The MST of the group targeted with PD-CD133/BSH in combination with BSH plus BNCT in CD133-glioma implanted mice or BSH plus BNCT in CD133+ glioma implanted mice was significantly different from untreated control group (*P* < 0.01) or the group of PD-CD133/BSH in combination with BSH plus BNCT in CD133+ glioma implanted mice (*P* < 0.05).

Before irradiation, mice were injected intratumorally with a solution of 5 μL PD-CD133/BSH for 12 h alone, or 100 mg/kg b.w. BSH i.v. for 1 h alone, or a combination. BNCT was performed using IHNI-1 21 days after mice were implanted with SU2 cells. All mice tolerant to BNCT were evaluated and processed as described in previous studies [11, 12]. Using the Kaplan–Meier survival analysis, we evaluated the survival times of glioma-bearing mice after BNCT. The survival data of mice implanted with SU2 cells are shown in Table 3, and Kaplan-Meier and Cox survival plots are displayed in Figure 7. In mice, not treated with neutron radiation, but only with CD133+ intracranial xenografts, the median survival time (MST) was 32.2 ± 4.9 days (95% CI, 29.2–35.2 days) for untreated control and 31.1 ± 3.1 days (95% CI, 28.6–33.6 days) for mice receiving PD-CD133/BSH in combination with BSH. In mice exposed to neutron radiation, the survival data varied significantly.

**Table 3:** Survival times of mice transplanted with CD133+ or CD133- glioma cell xenografts following treatment (d)

Group	Treatment	Range	Mean ± SE	Median
1	untreated control for CD133+ cells	24–39	32.2 ± 4.89	32
2	neutron radiation control for CD133+ cells	28–46	37.3 ± 6.41	34
3	PD-CD133/BSH + BSH for CD133+ cells	25–38	31.1 ± 3.14	30
4	BSH + BNCT for CD133+ cells	30–62	45.9 ± 9.08^**^	45
5	PD-CD133/BSH + BNCT for CD133+ cells	29–45	35.8 ± 4.60	34
6	PD-CD133/BSH + BSH + BNCT for CD133+ cells	40–> 90	61.8 ± 11.4^**^^#^^$^	57
7	PD-CD133/BSH + BSH + BNCT for CD133− cells	31–63	46.7 ± 9.36^**^	47

10 mice were included in each treatment group, sacrificed when symptoms of death appeared, and survival times were calculated according to the time between tumor transplantation and sacrifice. There was one mice surviving more than 90 days in group 6.

** *P* < 0.01 vs. group 1;

# *P* < 0.05 vs. group 4;

$ *P* < 0.05 vs. group 7.

Mice, which only received neutron radiation, had a MST of 37.3 ± 6.4 days (95% CI, 33.3–41.3 days). Mice bearing CD133+ SU2 gliomas, which received intratumoral injection of PD-CD133/BSH plus BNCT, also showed a MST of 35.8 ± 4.6 days (95% CI, 32.3–39.3 days). The MST of mice implanted with CD133+ SU2 gliomas followed by BSH plus BNCT was 45.9 ± 9.1 days (95% CI, 39.1–52.7 d). Mice that received PD-CD133/BSH combined with BSH with CD133+ SU2 gliomas showed a MST of 61.8 ± 11.4 days (95% CI, 52.8–70.8 d) with one mice surviving more than 90 days, but only 46.7 ± 9.4 days (95% CI, 39.8–53.6 days) in mice bearing CD133− SU2 glioma (Table 3).

Results indicate that following BNCT, the survival time in mice, receiving PD-CD133/BSH in combination with BSH, with CD133+ SU2 gliomas was significantly different from that of mice receiving PD-CD133/BSH in combination with BSH with CD133− SU2 gliomas (*P* < 0.05) and mice receiving BSH with CD133+ SU2 gliomas (*P* < 0.05). The differences in MSTs among the groups of untreated control, neutron radiation control, PD-CD133/BSH and PD-CD133/BSH plus BNCT were not significant (*P* > 0.05). However, statistically significant differences were noted in PD-CD133/BSH groups in combination with BSH plus BNCT in CD133- glioma implanted mice (*P* < 0.01) and BSH plus BNCT in CD133+ glioma implanted mice (*P* < 0.01) compared with untreated control. Survival in mice bearing brain tumors correlated significantly with BNCT by univariate analysis (*P* < 0.001). Multivariate analysis and Cox proportional hazards model indicated that BSH following BNCT was a predictor of poor prognosis (*P* < 0.001) for glioma-implanted mice (Figure 7).

### Histopathologic findings of the brains from glioma implanted mice following BNCT

The brains of mice in each group were perfomed histopathologic examination before sacrificed. Tumor sizes of mice that received BNCT after BSH alone, BSH plus PD-CD133/BSH injection for CD133+ cells, and PD-CD133/BSH plus BSH injection for CD133− cells xenograft were 2.5, 2.6 and 2.6 indices, respectively. Tumor sizes of mice in other groups including control, neutron irradiation alone, PD-CD133/BSH plus BSH, BNCT after PD-CD133/BSH injection were 2.0, 2.3, 2.1 and 2.3, respectively. The fact that these mice died with smaller tumors comparing to those treated with significant BNCT suggested that they probably had more peritumoral brain edema, which is considered to be one of the main biological behaviors of glioma and significantly associated with the clinical outcome of brain glioma.

**Figure 8:**
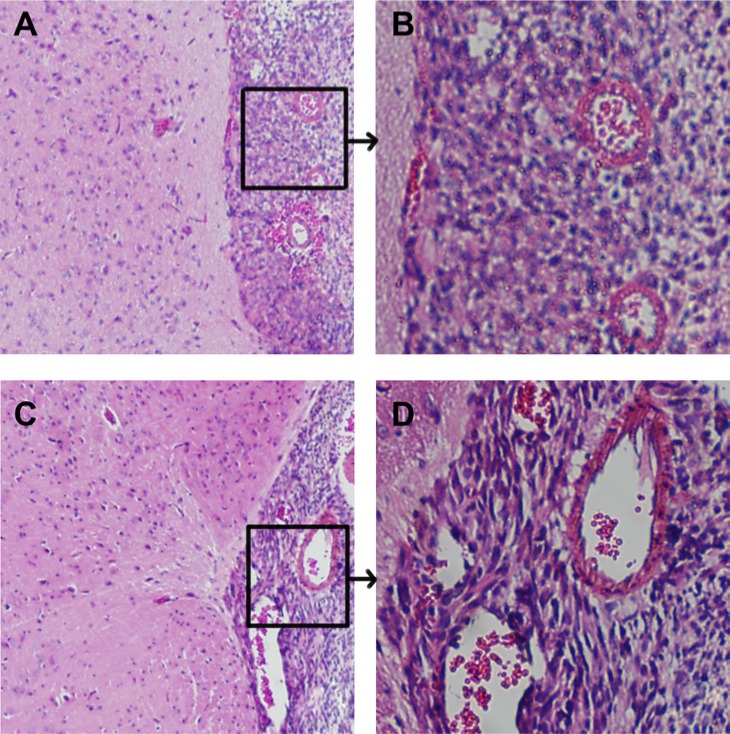
Histopathological characteristics of intracranial xenograft tumors Cellular pleomorphism including fusiform-shaped and small round tumor cells and highly proliferative nuclei were showed in tumor xenografts (**A**) Junction of tumor and brain tissue in control group, ×100, (**B**) karyokinesis phase in xenograft of control group, ×400, (**C**) Junction of tumor and brain tissue in PD-CD133/BSH in combination with BSH injection following BNCT for CD133+ cells group, ×100, (**D**) karyokinesis phase of xenograft in PD-CD133/BSH in combination with BSH injection following BNCT for CD133+ cells group, ×400.

Microscopically, histopathology of the HE stained sections of xenograft tumors in all mice demonstrated the replicating major histologic characteristics of clinical patients' GBM. There was considerable cellular and nuclear pleomorphism. Basic structure of normal brain tissue in the edema area was still preserved but the tissue was loose degree. The area of peritumoral brain was scattered invasive tumor cells, and the density of which was significantly higher in the area near compared to far from the glioma (Figure 8).

## DISCUSSION

The aim of this study was to develop a novel boron-based targeting agent, PD-CD133/BSH, and evaluate its role in sorting CD133+ U87s and SU2 GSCs using CD133 magnetic beads. The PAMAM dendrimers incorporated with CD133 antibody carry drugs, comprising BSH as a clinical BNCT agent encapsulated in PD-CD133 for high uptake by tumor cells.

The design of boron carriers in BNCT possesses a high potential for the increase of boron concentration in tumor cells [13]. BSH and boronophenylalanine (BPA) are the two boron-based drugs that are currently used in clinical trials of BNCT for patients with glioma [14]. In this study, we selected BSH for encapsulation in PAMAM dendrimers as the boron agent to enable the absorption of ^10^B atoms by targeted cells. The encapsulating efficiency and loading capacity of the bioconjugate PD-CD133 suggested that a large dose of BSH occupied the space inside the PAMAM dendrimers. The boron concentration was significantly higher when CD133+ cells were cultured with PD-CD133/BSH compared with BSH alone or for CD133− cells, which indicated that boron atoms accumulated in targeting cells following uptake of PD-CD133. Many recent results showed that a higher uptake of boron by tumor cells *in vitro* and *in vivo* when BSH was linked with biomaterials. Iquchi Y et al. created a BSH fused with a short arginine peptide-labeled positron emission tomography (PET) probe, and the imaging results showed a high uptake in the tumor area [15]. Genady AR and his colleagues compounded a series of mono- and dicarboxyalkyl BSH derivatives, and found that BSH-conjugated triazole 15 induced a significant increase in the level of boron accumulation in HeLa cells [16].

The concept of GSCs explains the limited efficacy of conventional chemo- and radiotherapy against GBM [17]. Effective eradication of GSCs by targeting CD133, a surface marker of GSCs, showed great potential to inhibit GBM re-growth in several experiments [18–20]. To facilitate increased BSH uptake in CD133+ GSCs in this study, a PAMAM dendrimer comprising site-specific conjugation with anti-CD133 monoclonal antibody (PD-CD133) containing BSH (PD-CD133/BSH) was synthesized to specifically target CD133+ cells. The performance of PD-CD133/BSH was evaluated *in vitro* using SU2 and U87s cells and *in vivo* using athymic mice with orthotopic glioma xenografts. Our results showed that more than 90% CD133+ cells absorbed PD-CD133/BSH compared with less than 40% CD133− cells *in vitro*. In animal models we also found similar uptake of PD-CD133/BSH to CD133+ cells. These results indicated that the targeted delivery of PD-CD133/BSH enhances its cellular uptake and improves therapeutic efficacy.

Greater tumor cell killing was observed as a function of increasing boron concentrations in some studies. A targeted multi-functional mesoporous silica nanoparticle, conjugated with trivalent galactosyl ligands and filled with carborane, was endocytosed by cells and released from lysosomes into the cellular cytoplasm. Further, compared with BSH, these nanoparticles provided a higher efficiency of delivery of boron atoms and a better effect of BNCT in neutron irradiation experiments [21]. High boron content and significant antitumor effect after thermal neutron irradiation were observed in mice injected with BSH-encapsulating 10% distearoyl boron lipid liposomes at a dose of 15 mg B/kg [22]. BNNT-DSPE-PEG2000 accumulated in B16 melanoma cells approximately three times higher than BSH, and the higher BNCT antitumor effect was observed in the cells treated with BNNT-DSPE-PEG2000 compared with those treated with BSH [23]. BSH-fused cell-membrane peptide permeated malignant glioma cells in a mouse brain tumor model. The administration of this compound showed a very high boron value and significant anti-cancer effect compared with the group administered with 100 times higher concentration of BSH [24]. In this study, the survival data with combination PD-CD133/BSH with BSH were superior to those administered single BSH in glioma implanted mice [13]. PD-CD133/BSH or BSH was given either alone or in combination, followed by BNCT. After neutron irradiation, the MST of mice with CD133 + SU2 glioma xenografts exposed to PD-CD133/BSH in combination with BSH was 61.8 ± 11.4 days, compared with 46.7 ± 9.4 days in mice implanted with CD133- SU2 glioma xenografts: 45.9 ± 9.1 days with BSH alone, and 35.8 ± 4.6 days with PD-CD133/BSH alone. In the group exposed to PD-CD133/BSH combined with BSH plus BNCT group, a single mouse survived > 90 days, which was regarded as prolonged survival. These results indicated that combination administration of PD-CD133/BSH and BSH following BNCT significantly extended survival time in glioma. A majority of glioma cells following uptake of BSH were killed after BNCT. In all tumor cells, a small number of CD133+ GSCs absorbing PD-CD133/BSH, were destroyed. After treatment, the GSCs which re-entered the proliferative phase were decreased, and the tumor recurrence was delayed. However, in some tumor cells, which absorbed neither BSH nor PD-CD133/BSH, CD133- GSCs were still alive after BNCT. This population played an important role in glioma recurrence. Our goal is to target this residual population with increased doses of boron in a future study.

The current preferential boron compound used in glioblastoma patients is L-boronophenylalanine (L-BPA), so our group will consider further research on the effect of the combination use of PD-CD133/BSH and L-BPA for targeting glioma stem cells. ^18^F-BPA was developed for positron emission tomography (PET) and is now an indispensable tool for the estimation of boron distribution in patients with tumor/normal tissue and tumor/blood ratios to determine the proper neutron doses before BNCT. The development of medical imageology of PD-CD133/BSH is needed if the agent would be used in clinical trials of BNCT. ^157^Gd-conjugating or magnescope-encapsulating PD-CD133/BSH can be a developing trend for boron detection in patients' GSCs, especially ^157^Gd neutron capture therapy also has the effect of killing cells [25]. These preliminary experiments showed that the PD-CD133/BSH can target CD133+ GSCs, indicating that this nanoparticle is a promising agent to be used to prevent regrowth of existing GBM. Whether the PD-CD133/BSH studied here can ultimately be used in clinical trials awaits further preclinical work to confirm that it can induce the regression of preexisting tumors in animal models.

In conclusion, the PD-CD133/BSH, a bioconjugate of PAMAM dendrimers linked with CD133 antibodies encapsulating BSH, showed significant superiority to CD133+ GSCs. A combination administration of PD-CD133/BSH and BSH following BNCT significantly extended the survival time of orthotopically transplanted tumor mice and was therapeutically effective against glioma.

## MATERIALS AND METHODS

### Synthesis of PD-CD133 biconjugate

**Figure 9:**
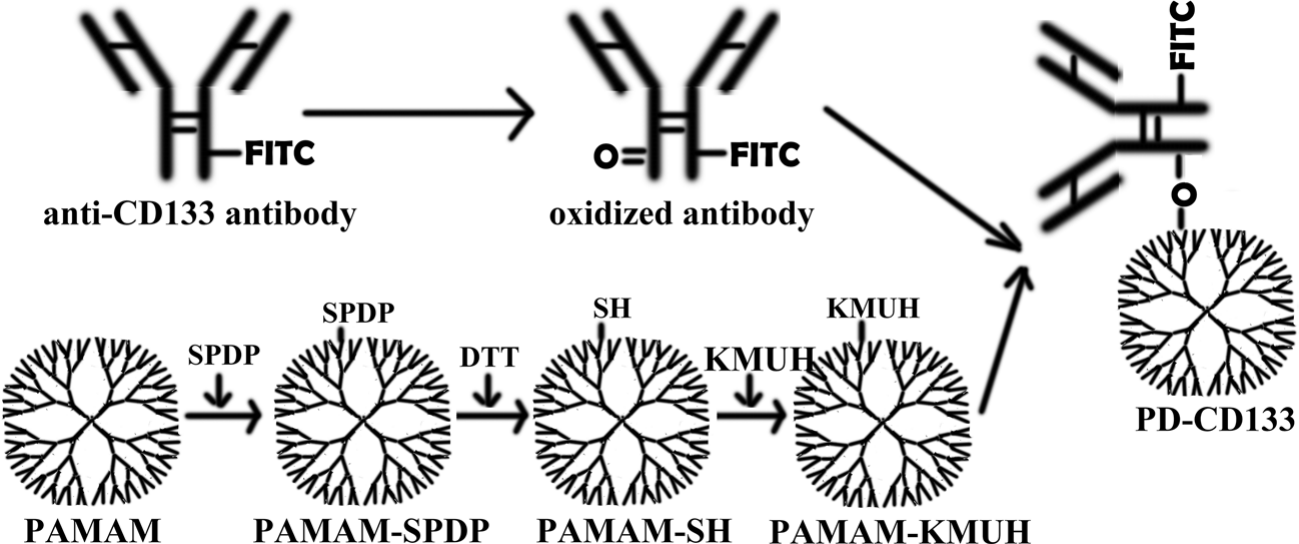
Preparation of a monoclonal antibody targeting nanoparticle A mAb-CD133 was oxygenated by NaIO4 to yield a oxygenated mAb-CD133. PAMAM dendrimer was conjugated to SPDP (PAMA-SPDP), the conjugate was deoxygenated by DTT to yield a sulfhydryl group (PAMAM-SH), then KMUH was linked to the synthetics containing sulfhydryl (PAMAM-KMUH). PAMAM-KMUH was conjugated to oxygenated mAb-CD133 to yield the biconjugate of PD-CD133.

A mAb-CD133 with a FITC group was linked to PAMAM dendrimers 5.0 generation (Sigma, St. Louis, MO) to yield PD-CD133 as described previously by Yang W [11, 12]. Briefly, mAb-CD133 was oxidized by NaIO4 in acetate buffer (pH 4.5). SPDP was conjugated to PAMAM dendrimers, and the disulfide bond was subsequently cleaved by DTT to yield a sulfhydryl group. KMUH was conjugated to sulfhydryl-containing synthetics (PAMAM-SH), linked to oxidized mAb-CD133 to yield the PD-CD133 biconjugate (Figure 9). All low molecular weight contaminants were removed by ultrafiltration (MWCO, 10K), and the final biconjugate was purified by a Sephacryl S-300 gel filtration column. The absorbance of the biconjugated product was detected using ultraviolet-visible spectra to ensure conjugation of mAb-CD133 and PAMAM. The purity was determined by SDS-PAGE stained with Coomassie Brilliant Blue. The average number of mAb-CD133 conjugated to the dendrimer was determined using the Ellman assay. A standard curve was plotted with free antibodies. The absorbance at 412 nm was used to calculate the number of antibodies conjugated to the dendrimer according to the standard curve. Protein concentrations of biconjugate were read at 280 nm using a multiscan spectrophotometer 1510 (Thermo Fisher Scientific, Vantaa, Finland).

### Encapsulating efficiency and drug loading capacity

The EE% and LC% of BSH in PAMAM and PD-CD133 was determined by measuring boron concentration. In brief, ^10^B-enriched BSH was added to PAMAM and PD-CD133 solution, and unencapsulated BSH was separated by gel filtration chromatography. ^10^B in unencapsulated BSH, PAMAM encapsulating BSH (PAMAM/BSH) and PD-CD133 encapsulating BSH (PD-CD133/BSH) were detected using inductively coupled plasma atomic emission spectrophotometry (ICP-AES). The EE% and LC% were calculated as indicated below (*n* = 3). EE% = (BSH in PAMAM or PD-CD133/total amount of BSH in solution) × 100%, LC% = (BSH in PAMAM or PD-CD133 / solutes total weight) × 100%. Preparation of PD-CD133/BSH was detected by ultraviolet-visible spectra.

### PD-CD133/BSH uptake in surgical samples of glioblastoma

GBM tissues (grade IV) were collected immediately after excision from 3 patients. PD-CD133/BSH uptake assay was conducted as previously described [26]. GBM tissues were incubated with 0.1 μM PD-CD133/BSH for 12 h. The prepared frozen sections were examined for uptake of biconjugate nanoparticles using fluorescence microscopy.

CD133 protein expression in the tumors was analyzed by immunofluorescence. After blocking in 1% bovine serum albumin for 30 min at 37°C, tumor sections were incubated at 4°C overnight with anti–human CD133 mouse antibody (1:50, Miltenyi Biotech GmbH) diluted with the blocking buffer in a humidified chamber. The sections were incubated with a secondary antibody (1:500) conjugated to Alexa 555 (Invitrogen) for 1 h at room temperature. Slides were counter-stained with 4′,6-diamidino-2-phenylindole (DAPI, Southern Biotech). Fluorescent microscopy (Olympus BX50, Japan) of the three stained tissue sections was performed. The number of positively stained cells in each section was counted in 10 microscopic fields (at 400 × magnification), and the mean percentage per visual field was calculated.

### Cell culture

SU2 cells, representing a stem cell line of Chinese glioma origin, isolated from a 52-year-old female patient who had undergone two operations in a 6-month interval diagnosed with recurrent GBM (WHO Grade IV) of the right temporal lobe, were obtained as a gift from Professor Qiang Huang and described in detail elsewhere [27]. The human glioma cell line U87 was purchased from the Cell Bank Type Culture Collection of the Chinese Academy of Sciences (Shanghai, China) and cultured in DMEM/F12 medium (1:1, Hyclone) supplemented with 10% fetal bovine serum. GSCs U87s were cultured using a serum-free cloning technique as SU2 cells, and neurospheres of 12 passages were used for sorting [26]. SU2 and U87s cells were cultured in serum-free DMEM/F12 (1:1) (SIGMA-Aldrich, Tokyo, Japan) containing human recombinant N2 (1%), EGF and bFGF (20 ng/mL; Invitrogen, California, U.S.), and maintained at 37°C and 5% CO_2_ in a humidified incubator.

### CD133+ cell enrichment and flow cytometry

Magnetic isolation of SU2 and U87s cells was carried out using the Miltenyi Biotec CD133 Cell Isolation kit (Miltenyi Biotec GmbH). The sorted CD133+ and CD133− cell populations were isolated by magnetic beads. Flow cytometry was performed using a flow cytometer Beckton Dickinson FACScan (BD Biosciences, San Jose, CA) to analyze the percentage of CD133+ cells.

### Immunofluoresence staining of stem cell markers

Neurospheres were digested into single-cell suspensions and dropped on microslides treated with poly-L-lysine, rinsed with sterile water, sterilized with UV and dried. After fixing for 15 min with 4% paraformaldehyde at room temperature, cells were blocked with 5% bovine serum albumin and permeabilized with 0.1% Triton X-100. Primary mouse antibodies against human CD133 (1:100) and nestin (1:100, Abcam) were added and incubated overnight at 4°C. Fluorescence-labeled secondary antibodies were added and incubated at room temperature for 1 h in darkness. The cells were counterstained with DAPI and analyzed by fluorescence microscopy.

### Uptake of PD-CD133/BSH *in vitro*

The 2 × 10^5^ CD133+ or CD133- cells suspended in 2 mL nutrient medium were seeded in 6-well plates for 24 h. To compare the difference in uptake efficiency between CD133+ and CD133− cells, the two cell lines were incubated with PD-CD133/BSH at different concentrations ranging from 0.05 to 1.0 μM for 12 h or different intervals ranging from 1.0 to 24.0 h to examine the optimal uptake dosage and time. The cells were washed twice with PBS, digested into single-cell suspensions and counterstained with DAPI, and observed directly under fluorescence microscope at an excitation wavelength of 488 nm. The uptake efficiency was expressed as the percentage of FITC-positive cells.

### Detection of boron accumulation

The 2 × 10^5^ CD133+ or CD133− cells were incubated with 0.1 μM PD-CD133/BSH solution (containing 2.2 μM BSH) or 2.2 μM BSH for 12 h. BSH was solved in PBS. The cells were washed twice with a phosphate buffer solution (PBS), and digested at 120°C in 1 mL of a mixture of nitric acid and hydrogen peroxide (3:1 v/v) for 2 h, until the solution was transparent. Deionized water was then added to 5 mL before estimating boron using ICP-AES.

### Neutron irradiation

In-Hospital Neutron Irradiator (IHNI-1) reactor with a maximum capacity of 30 KW and a thermal neutron flux of 1 × 10^9^ n/(cm^2^·s), was used for irradiation experiments. The 2 × 10^5^ SU2 or U87s cells were incubated with 2.2 μM BSH or 0.1 μM PD-CD133/BSH solution for 12 h, then washed twice with PBS, followed by irradiation experiments. The IHNI-1 components are displayed and the calculated irradiation doses are shown in Table 4.

**Table 4:** Radiation dosages in various groups of SU2 and U87s cells (Gy)

Cell Lines	Group		Time (min)					
			5	10	15	20	25	30
SU2	Neutron radiation (CD133+ cells)	^10^B(n,α)^7^Li	——	——	——	——	——	——
		total dosage	0.083	0.166	0.249	0.332	0.415	0.498
	BSH (CD133+ cells)	^10^B(n,α)^7^Li	0.047	0.094	0.141	0.188	0.235	0.282
		total dosage	0.130	0.260	0.390	0.520	0.650	0.780
	PD-CD133/BSH (CD133− cells)	^10^B(n,α)^7^Li	0.111	0.222	0.333	0.444	0.555	0.666
		total dosage	0.194	0.388	0.582	0.776	0.970	1.164
	PD-CD133/BSH (CD133+ cells)	^10^B(n,α)^7^Li	0.503	1.006	1.509	2.012	2.515	3.018
		total dosage	0.586	1.172	1.758	2.344	2.930	3.516
U87s	Neutron radiation (CD133+ cells)	^10^B(n,α)^7^Li	——	——	——	——	——	——
		total dosage	0.083	0.166	0.249	0.332	0.415	0.498
	BSH (CD133+ cells)	^10^B(n,α)^7^Li	0.041	0.082	0.123	0.164	0.205	0.246
		total dosage	0.124	0.248	0.372	0.496	0.620	0.744
	PD-CD133/BSH (CD133− cells)	^10^B(n,α)^7^Li	0.105	0.210	0.315	0.420	0.525	0.630
		total dosage	0.188	0.376	0.564	0.752	0.940	1.128
	PD-CD133/BSH (CD133+ cells)	^10^B(n,α)^7^Li	0.486	0.972	1.458	1.944	2.430	2.916
		total dosage	0.569	1.138	1.707	2.276	2.845	3.414

The physical dose of the radiobiologically significant beam components include contributions from ^10^B(n,α)^7^Li, ^1^H(n,n')p, ^14^N(n,p)^14^C, and total γ (beam and ^1^H(n,γ)^2^H).

### Clonogenic survival assay

Cells were grown in 6-well plates at a density of 200–1000 cells per well and incubated for up to 3 weeks after irradiation. Colonies were fixed with 50% ethanol and stained with 5% crystal violet. The colonies with more than 50 cells were counted and the survival fractions were calculated based on the survival of non-irradiated cells. The valueof surviving fraction at different radiation dose was normalized to that of the same group at 0 Gy.

### Intracranial transplantation of GBM xenografts

All animal experimental protocols were approved by the Institutional Animal Care and Use Committee of Suzhou University and complied with the code of ethics for animal experiments. Four-week-old male BALB/c nude mice (18–20 g) were bred and maintained in the Specific Pathogen-Free Animal Care Facility. The 10^5^ sorted CD133+ or CD133− SU2 cells were injected into the right caudate nucleus using a stereotactic implantation apparatus to establish xenografts. The mice were used for the following experiment^s.^

### Targeting of PD-CD133/BSH *in vivo*

Mice with CD133+ SU2 cellular xenografts received an intratumoral injection of 5 μL PD-CD133/BSH (3.5 μg ^10^B) solution for 1 min using microsyringe directly, and were sacrificed about 12 h later. Prior to the collection of mouse brains bearing GBM tumors, cardiac perfusion with PBS followed by 4% paraformaldehyde (PFA) was performed. The whole brain was harvested and continuously sectioned at a thickness of 7 μm. The CD133 protein was stained by immunofluorescence in the tumors concurrently. The slides were observed under fluorescent microscopy.

### Biodistribution of ^10^B in BALB/c nude mice with glioma xenografts

Glioma xenografts with CD133+ or CD133− SU2 cells in the mouse brain were injected with 5 μL PD-CD133/BSH solution *in situ*. After 11 h, BSH was injected at a dose of 100 mg/kg b.w. into the tail vein of nude mice, which were untreated or pretreated with PD-CD133/BSH. Animals were killed at 1 h after BSH injection [14]. Samples of blood, contralateral normal brain and tumor tissue from all the mice were collected. The boron determination was performed with ICP-AES, and the boron concentrations were normalized to 1 g weight of the tissue sample.

### BNCT experiments of mice bearing glioma

Mice were randomly divided into 7 experimental groups and 10 mice were in each group: Group 1, control; group 2, neutron irradiation control; group 3, intratumoral injection of PD-CD133/BSH plus i.v. BSH; group 4, i.v. BSH and BNCT; group 5, intratumoral injection of PD-CD133/BSH and BNCT; group 6, intratumoral injection of PD-CD133/BSH plus i.v. BSH and BNCT; group 7, intratumoral injection of PD-CD133/BSH plus i.v. BSH and BNCT. CD133+ SU2 cells were implanted with intracranial xenografts in groups 1 to 6, and CD133− cells were used in group 7. BNCT was conducted 21 days after SU2 cell implantation using IHNI-1 as the neutron source. BNCT was performed at 12 h after intratumoral injection of PD-CD133/BSH (3.5 μg ^10^B/5 μL) and 1 h after i.v. administration of 100 mg/kg BSH. A ^6^Li-enriched polyethylene box was used to shield the whole body of mice from thermal neutrons during irradiation, and the head of each mouse was exposed to neutron irradiation. Animal irradiations were conducted with IHNI-1, and dosimetric calculations of irradiation including γ-photons, ^14^N(n,p)^14^C and ^10^B(n,α) ^7^Li reactions were based on mean boron concentrations of tumor, normal brain, and blood at 12 h after intratumoral administration of PD-CD133/BSH or 1 h after i.v. administration of BSH in mice with glioma xenografts (Table 5).

**Table 5:** Radiation dosage of tumors, contralateral normal brain and blood (Gy)

Glioma xenografts	Group	Tumor	Ipsilateral brain	Contralateral brain	Blood	percent (%)
CD133+ cells	BSH + BNCT	8.3	6.7	6.2	5.5	108.1
	PD-CD133/BSH + BNCT	5.1	3.5	3.5	3.3	100.0
	PD-CD133/BSH + BSH + BNCT	9.3	7.0	6.3	5.5	111.1
CD133− cells	PD-CD133/BSH + BSH + BNCT	8.6	6.8	6.3	5.4	107.9

Absorbed dose determinations included contributions from ^10^B(n,α)^7^Li, ^1^H(n,n')p, ^14^N(n,p)^14^C, and total γ (beam and ^1^H(n,γ)^2^H). The percent (%) was calculated according to radiation dosage of ipsilateral vs. contralateral brain.

### Monitoring of brain tumor status and statistical analysis of survival

The condition of the mice was monitored daily for brain tumor symptoms, and weighed three times a week. Common symptoms such as sustained weight loss, ataxia and periorbital bleeding, or a combination thereof, indicated imminent death. When symptoms of death appeared, mice were euthanized and survival times were calculated according to the time between tumor transplantation and euthanization. Mice surviving more than 3 months were designated long-term survivors and were euthanized. The mean survival time (MST) and median survival time were calculated for each group.

### Statistical analysis

All analyses were performed from at least 3 independent experiments as indicated, using SPSS 10.0 for Windows (SPSS Inc., Chicago, IL, USA). The data were presented as the mean ± SE. Statistically significant differences between the 2 groups were determined with Student's *t* test. Data were considered statistically significant at *P* < 0.05. Survival of glioma bearing mice was calculated for each group using the Kaplan–Meier estimate, the log-rank test was used to test for significance between survival curves. A Cox proportional hazards regression model was suitable for analyzing the survival data, and the Wald test was used for these comparisons, with a Bonferroni method of adjustment for the multiple comparisons.
